# Trophic Status Is Associated With Community Structure and Metabolic Potential of Planktonic Microbiota in Plateau Lakes

**DOI:** 10.3389/fmicb.2019.02560

**Published:** 2019-11-07

**Authors:** Mengyuan Shen, Qi Li, Minglei Ren, Yan Lin, Juanping Wang, Li Chen, Tao Li, Jindong Zhao

**Affiliations:** ^1^State Key Laboratory of Freshwater Ecology and Biotechnology, Institute of Hydrobiology, Chinese Academy of Sciences, Wuhan, China; ^2^University of Chinese Academy of Sciences, Beijing, China; ^3^State Key Laboratory of Lake Science and Environment, Nanjing Institute of Geography and Limnology, Chinese Academy of Sciences, Nanjing, China; ^4^Yunnan Key Laboratory of Plateau Geographical Processes and Environment Change, School of Tourism and Geography, Yunnan Normal University, Kunming, China; ^5^State Key Laboratory of Protein and Plant Genetic Engineering, College of Life Sciences, Peking University, Beijing, China

**Keywords:** metagenomics, trophic status, taxonomic diversity, community structure, metabolic potential, planktonic microbiota, lake ecosystem, Cyanobacterial bloom

## Abstract

Microbes in various aquatic ecosystems play a key role in global energy fluxes and biogeochemical processes. However, the detailed patterns on the functional structure and the metabolic potential of microbial communities in freshwater lakes with different trophic status remain to be understood. We employed a metagenomics workflow to analyze the correlations between trophic status and planktonic microbiota in freshwater lakes on Yun-Gui Plateau, China. Our results revealed that microbial communities in the eutrophic and mesotrophic-oligotrophic lake ecosystems harbor distinct community structure and metabolic potential. Cyanobacteria were dominant in the eutrophic ecosystems, mainly driving the processes of aerobic respiration, fermentation, nitrogen assimilation, nitrogen mineralization, assimilatory sulfate reduction and sulfur mineralization in this ecosystem group. Actinobacteria, Proteobacteria (Alpha-, Beta-, and Gammaproteobacteria), Verrucomicrobia and Planctomycetes, occurred more often in the mesotrophic-oligotrophic ecosystems than those in the eutrophic ecosystems, and these taxa potentially mediate the above metabolic processes. In these two groups of ecosystems, a difference in the abundance of functional genes involved in carbohydrate metabolism, energy metabolism, glycan biosynthesis and metabolism, and metabolism of cofactors and vitamins significantly contribute to the distinct functional structure of microbiota from surface water. Furthermore, the microbe-mediated metabolic potentials for carbon, nitrogen and sulfur transformation showed differences in the two ecosystem groups. Compared with the mesotrophic-oligotrophic ecosystems, planktonic microbial communities in the eutrophic ecosystems showed higher potential for aerobic carbon fixation, fermentation, methanogenesis, anammox, denitrification, and sulfur mineralization, but they showed lower potential for aerobic respiration, CO oxidation, nitrogen fixation, and assimilatory sulfate reduction. This study offers insights into the relationships of trophic status to planktonic microbial community structure and its metabolic potential, and identifies the main taxa responsible for the biogeochemical cycles of carbon, nitrogen and sulfur in freshwater lake environments.

## Introduction

The microbiota in aquatic ecosystems plays an important role in elemental cycling and global energy fluxes (Falkowski et al., 2008; Clark et al., 2018; Cronan, 2018). The relations between the taxonomic structure of microbial communities in aquatic environments and complex environmental factors such as trophic status (Llirós et al., 2014; Wan et al., 2017), seasons (Zhu et al., 2019), elevation gradient (Li H. et al., 2017), and salinity (Eiler et al., 2014) have been well studied. However, little is known about the correlations of these factors with community functions. Therefore, improving our knowledge about the link between taxonomy and function of microbial communities can contribute to a better understanding of the response mechanisms of microbiota to key environmental changes and gradients (Logue et al., 2015; Arora-Williams et al., 2018).

The Yun-Gui Plateau Lake Zone is the smallest of the five lake-zones in China (Ma et al., 2011). About half of the lakes in this zone, accounting for 90% of the lake area, are located in Yunnan Province which is a biodiversity hotspot (Zhou et al., 2019), and these lakes are sensitive areas for recording regional ecology and global climate change (Li et al., 2015). The plateau lake ecosystems are vulnerable and not easily restored once damaged because of the relatively low rate of water exchange and resilience, and the steep and little-developed lakeshores (Wang and Dong, 1998; Liao et al., 2016). In the past few decades, some of these lakes have been seriously damaged by intensification of human activities, leading to deterioration of water quality and degradation of ecosystem function (Li W. et al., 2017; Liu et al., 2017; Gao et al., 2018; Wu et al., 2019). Eutrophication is one of the biggest of such challenges; it changes the diversity and composition of lake organisms and poses a serious threat to ecosystem service function (Liu et al., 2012; Shi et al., 2016; Dong et al., 2018). To date, most studies have concentrated on microbial communities in sediment from Yun-Gui Plateau lakes with different trophic levels (Bai et al., 2012; Dai et al., 2016; Yang et al., 2017a, b). Only a few studies have focused on microbiota in lake surface waters, in which the microorganisms are more sensitive to lake eutrophication than those in sediment (Zeng et al., 2019). Bacterioplankton compositions in eutrophic Lake Dianchi (Wen et al., 2012; Dai et al., 2016; Han et al., 2016), mesotrophic Lake Erhai (Hu et al., 2013) and oligotrophic Lake Haixihai (Dai et al., 2016) were investigated by analyzing 16S rRNA gene sequences. Dai et al. (2016) and Han et al. (2016) demonstrated that trophic status may play important roles in shaping the taxonomic structure of bacterioplankton communities in the Yun-Gui Plateau freshwater lakes. Nevertheless, the relations of lake trophic status to the functional structure of the microbial communities and the ecological processes within freshwater systems have seldom been examined.

Because of decreased cost and increased throughput of sequencing technology (Neufeld, 2017; Quince et al., 2017), the powerful approach of metagenomics is now widely applied in studies of microbial communities from many diverse environments, including soil (Diamond et al., 2019), sediment (Vavourakis et al., 2018), hosts (Rothschild et al., 2018), seawater (Sunagawa et al., 2015), and freshwater (Arora-Williams et al., 2018). A curated set of metabolic marker genes was used to quantify the genetic potential for microbe-mediated biogeochemical cycles in a meromictic lake by Lauro et al. (2011). This method has since been widely used in different types of ecosystem, including salt marsh (Dini-Andreote et al., 2016), sediments (Hamilton et al., 2016), an estuary (Kieft et al., 2018), and a stratified euxinic lake (Llorens-Marès et al., 2015). Therefore, besides the characterization of community structure and reconstruction of genomes in individual samples, comparative analysis of the samples at multiple time points or of parallel samples across different environmental gradients using metagenomics facilitates the elucidation of complex microbial processes in the community, which are difficult to simulate in the laboratory.

In this study, we applied shotgun metagenomics to examine the taxonomic and functional structure of surface-water microbial communities from five freshwater lakes on the Yun-Gui Plateau. These lakes had four trophic levels: eutrophic, meso-eutrophic, oligo-mesotrophic and oligotrophic. The relative abundance of metabolic marker genes was used to assess the genetic potential for each conversion step of the carbon, nitrogen, and sulfur cycles in the freshwater lake ecosystems. We explored the links between microbial composition and metabolic potential, and inferred the response mechanisms of microbe-mediated carbon, nitrogen, and sulfur cycles to lake trophic-level changes. We addressed the following two questions: (a) How does trophic status relate to distinct taxonomic and functional structures of planktonic microbial communities? (b) To what extent is it related to trophic status that each conversion step of microbe-mediated biogeochemical cycling pathways?

## Materials and Methods

### Study Sites and Sampling

To investigate the relationship of trophic status and the microbial communities in a plateau lake ecosystem, five lakes with different trophic status were selected in Yunnan Province, China (Supplementary Figure S1 and Table 1). Dianchi Lake (DCL) and Xingyun Lake (XYL) are eutrophic lakes, turbid with abundant algae (Yang et al., 2010; Gao et al., 2018). Erhai Lake (EL) has undergone alteration from mesotrophic to eutrophic conditions owing to excessive usage of chemical fertilizers and severe destruction of wetland vegetation along the lakeshore (Hu et al., 2014; Wang et al., 2015a). Fuxian Lake (FXL) is oligo-mesotrophic (Cui et al., 2008). Lugu Lake (LGL) is oligotrophic, and clear with abundant submerged plants (Liao et al., 2015). Water samples were collected from the surface layer (0–0.5 m depth) of each lake between June 2014 and September 2017. Our study focused on the planktonic microbes in water samples, and the methods of sample collection are described in Supplementary Table S1 and Supplementary Figure S2. Two samples (DCL-1 and XYL-1) from eutrophic lakes during the algal bloom period were filtered to enrich the “Cyanobacteria-attached” fraction (CA, >64 μm) (Li et al., 2011), and the other samples were collected with mixed-size fractions (>0 μm, >0.2 μm, and 0.2–64 μm). The volume of water sampling water was determined by the abundance of the planktonic microbial community and the size-fraction of filtration to ensure sufficient biomass for metagenomic DNA extraction. Collected biomass was stored at −80°C until processing. The sampling dates, sampling locations and physicochemical properties of lake water are shown in Table 1. A flow diagram describing the data analysis process is shown in Supplementary Figure S3, and scripts used in this study are available in a public GitHub repository^1^.

**TABLE 1:** Description of the samples used in this study.

**Sample ID**		**DCL-1**	**DCL-2**	**XYL-1**	**XYL-2**	**EL-1**	**EL-2**	**FXL-1**	**FXL-2**	**LGL-1**	**LGL-2**
Physicochemical properties	Date	20160618	20170925	20160607	20170924	20140614	20170721	20151210	20170924	20160518	20170812
	Location	24.96°N	24.95°N	24.38°N	24.36°N	25.94°N	25.90°N	24.57°N	24.38°N	27.71°N	27.71-27.73°N
		102.65°E	102.66°E	102.78°E	102.79°E	100.16°E	100.15°E	102.89°E	102.85°E	100.78°E	100.76-100.80°E
	WT (°C)	22.13	21.80	25.08	23.80	23.50	22.20	16.91	22.40	15.19	20.87
	PH	8.45	8.63	8.88	9.45	9.51	8.66	8.04	8.94	8.18	8.76
	TP (mg/L)	0.544	0.351	0.468	0.581	0.032^∗^	0.031	0.022	0.020	0.024	0.013
	TN (mg/L)	5.815	4.816	5.181	4.151	0.57^∗^	0.593	0.220	0.168	0.048	<0.103
Geographic information#	Lake	Lake Dianchi		Lake Xingyun		Lake Erhai		Lake Fuxian		Lake Lugu	
	Trophic status	Eutrophic		Eutrophic		Mesoeutrophic		Oligomesotrophic		Oligotrophic	
	Basin	The Yangtze River		The Pearl River		The Lancang River, Jinsha, and Yuanjiang Rivers		The Pearl River		The Yangtze River	
	Water level (m a.s.l.)	1887.4		1722		1971		1721		2690.75	
	Area (km^2^)	308.6		34.7		249.8		211		48.25	
	Average water depth (m)	4.4		7		10.5		87		40.3	
	Maximum depth(m)	6		11		21.5		155		93.5	
	Volume (10^8^ m^3^)	11.69		1.84		25.31		189		19.53	
	Lake type	Shallow		Shallow		Shallow		Deep		Deep	
Metagenomic survey	Clean data (Gbps)	50.60	58.91	50.38	64.12	63.43	63.98	60.49	53.01	51.85	41.43
	Number of clean reads	337,333,394	392,760,680	335,888,304	427,445,344	422,842,886	426,528,358	403,286,044	353,402,296	345,656,552	276,184,372
	Number of contigs (>500 bps)	2,381,193		2,318,367		3,929,297		3,368,043		1,604,121	
	Number of predicted CDS	3,775,712		3,059,812		6,560,747		5,229,770		2,840,196	
	% Predicted CDS with taxonomic group assignment	60.22		44.12		57.69		40.85		58.89	
	% Predicted CDS with KOs assignment	26.45		20.66		27.24		20.23		27.91	
	Bacteria (%, based on reads from metagenomic data)	23.31	33.11	14.66	41.15	13.65	18.79	19.69	12.55	22.61	28.97
	Archaea (%, based on reads from metagenomic data)	0.04	0.02	0.01	0.01	0.02	0.03	0.03	0.02	0.03	0.02
	Viruses (%, based on reads from metagenomic data)	0.74	0.09	0.03	0.03	0.06	0.14	0.28	0.08	0.39	0.11

*TN, total nitrogen concentration (mg/L); TP, total phosphorus concentration (mg/L); WT, water temperature; ^∗^Data source: Cao et al. (2018). ^#^Data source: [opetwcite]B12,B86[clotwcite]Cui et al. (2008); Yang et al. (2010), [opetwcite]B29,B45[clotwcite]Hu et al. (2014); Liao et al. (2015), Wang et al. (2015a, b), [opetwcite]B15,B22[clotwcite]Ding et al. (2017); Gao et al. (2018).*

### DNA Extraction, Sequencing, and Assembly

Total community DNA extraction was conducted following a modified phenol-chloroform method from Xie et al. (2016). Metagenome sequencing was performed on an Illumina Genome Analyzer IIx, and yielded > 40 GB per library (>276 M reads, 150 bp paired-end, insert size ∼300 bp). Reads from the same lake were then co-assembled with MEGAHIT assembler (Li et al., 2016) (v.1.1.1, with preset meta-large). Coding sequences (CDSs) were predicted using Prodigal v2.6.3 (-p meta) in all contigs > 500 bp long (Hyatt et al., 2012). In each sample, clean reads were aligned back to the contigs using Bowtie2 (Langmead and Salzberg, 2012) and counted by featureCounts (Smyth et al., 2013). The number of reads for each CDS was normalized to “transcripts per million” (TPM) as described elsewhere (Ribicic et al., 2018).

### Taxonomic and Functional Assignment of Metagenome

Two main approaches were chosen for taxonomic annotation, involving assembly free and assembly based methods (Quince et al., 2017). The clean reads from each sample were classified for community composition analysis by using the assembly free approach. The Kaiju classifier (Menzel et al., 2016) was used to assign metagenomic reads against the subset of NCBI-NR protein database (bacteria, archaea, virus) (*E*-value 0.05). Then, the kaijuReport program was used to count the phylum-level, class-level, and order-level abundance of each sample. In addition, the predicted CDSs were taxonomically assigned to filter eukaryotic contamination by using the assembly based method. Diamond (Buchfink et al., 2015) was used to compare predicted protein sequences against the NR database (version Apr 2, 2019; blastp -f 100 -e 0.00001 –sensitive –top 3), and then the LCA algorithm in MEGAN6 (blast2lca -f DAA -m BlastP) was performed for CDSs to produce a taxonomic classification (Huson et al., 2016).

Functional analysis was performed based on microbial CDSs. The GhostKoala server^2^ was used to functionally annotate each CDS by giving KEGG Orthology (KO) accession numbers (Kanehisa et al., 2016). Then, the annotated functional CDSs were extracted and assigned to KEGG metabolism of level 2 categories for subsequent distribution analysis of CDSs with metabolism. The TPM values of CDSs from the same functional category were added together.

The analysis of metabolic potential focused on three elemental biogeochemical cycles (carbon, nitrogen, and sulfur) for the four trophic lake types. To infer the genetic potential of each lake ecosystem, the relative abundance of metabolic marker genes (KO accession numbers) identified in previous studies was calculated as described elsewhere (Lauro et al., 2011; Llorens-Marès et al., 2015; Dini-Andreote et al., 2016; Hamilton et al., 2016; Kieft et al., 2018). In this study, 50 marker genes were used, representing 20 microbe-mediated elemental cycling processes (Supplementary Table S2).

### Statistical Analysis

Phylum-level read count data matrices and functional abundance matrices were Hellinger-transformed, respectively. Unweighted pair group method with arithmetic mean (UPGMA) clustering analysis and principal coordinate analysis (PCoA) were used to display and compare the patterns of taxonomic structure and metabolic function among different samples. In addition, the significant variance (*P* < 0.01) between groups of samples was assessed by permutational multivariate analysis of variance (PERMANOVA). The correlation between taxonomic and functional composition was calculated by the Mantel test (9999 permutations). Similarity percentage (SIMPER) analysis determined the contributions from each metabolic function group to PERMANOVA reported differences. Based on the taxonomic annotation of the metagenomic reads results at the order-level, the alpha-diversity of each community was calculated using the ‘diversity()’ function.

Environmental data were normalized to *z*-scores before calculating distance. Euclidean distance was used for environmental data, and Bray-Curtis distance was used for compositional data. Based on these distance matrices, Mantel correlations between environmental data and taxonomic and functional compositional data were calculated, respectively. Furthermore, pairwise Pearson’s correlation analysis was carried out to examine the relationship between environmental variables. Pearson’s correlation analyses between all environmental factors and the relative abundances of the functional categories were performed using the ‘corr.test’ function. Redundancy analysis (RDA) was performed to investigate the relationships between environmental variables and microbial communities. Based on Monte Carlo permutation tests (*n* = 999 permutations), only the significant environmental variables were accepted (*p* < 0.05) for RDA. In addition, to avoid col-linearity among environmental variables, high variance inflation factors (VIF > 20) were eliminated. Environmental variables significantly explaining community variations were selected by using forward model selection with the ‘ordistep’ function, and then the variance explained by each key variable was evaluated by variation partitioning. All of the above statistical analyses were performed using the vegan (Oksanen et al., 2019) and psych (Revelle, 2019) packages in R version 3.5.3.

STAMP software was used to test for differences in microbial community structure and relative abundance of KOs between groups, as described elsewhere (Castro-Nallar et al., 2015). White’s non-parametric *t*-test in STAMP was applied to compare the relative abundance of phyla, orders and KOs between two groups of lake samples (White et al., 2009). A percentile bootstrapping method (10,000 replications) was used to estimated confidence intervals, and the false discovery rate (FDR) in multiple testing was corrected with the Storey’s FDR method (*p* < 0.05) (Storey et al., 2004). The trophic preference KO lists were then uploaded to the online functional pathway mapping tool iPath3 (Interactive Pathways Explorer v3^3^) for visualization. KOs that differentially segregated across groups were identified from 50 metabolic marker genes by random forest analysis with Boruta feature selection (R package Boruta, maxRuns = 1000).

## Results

### Diversity of Microbial Communities

On average, 372.13 million high-quality sequence reads with an average length of 150 bp were obtained from 10 samples from the five lakes with different trophic levels located on the Yun-Gui Plateau (Table 1). The taxonomic assignment of the microbial communities was performed using short reads-based methods. A minority of metagenomic reads could be classified (12–41%). The bacterial domain was the main taxonomic component of the microbial community in all lake samples.

**FIGURE 1:**
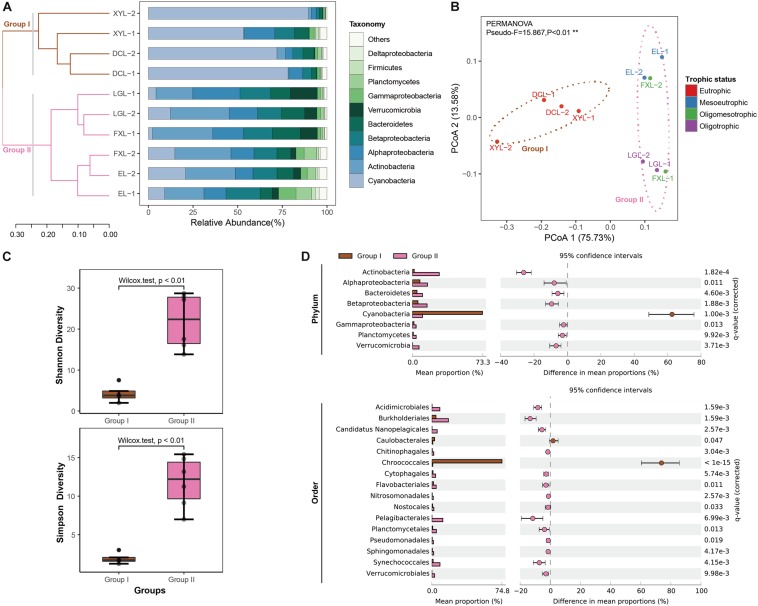
Taxonomic structure and diversity of planktonic microbial communities in lakes exhibiting different trophic status. **(A)** The taxonomic structure across samples. The relative abundance of reads grouped at the phylum-level is shown for each metagenome library. Phyla with relative abundance not in the top ten are shown as “Other.” Hierarchical clustering (UPGMA) based on Bray–Curtis dissimilarity matrices. See detailed information in Supplementary Table S4a. **(B)** PCoA based on complete taxonomic community profiles with 75% confidence ellipses (phylum-level taxonomic annotations). Significant clusters are indicated by dashed lines (PERMANOVA, 9999 permutations, *P* < 0.01). **(C)** Boxplots figure shows the range of different alpha diversity indices. The box represents the lower quartile, median, and upper quartile. See detailed information in Supplementary Table S3. **(D)** The extended error bar plot shows that phyla and orders significantly over−⁣/⁣−under-represented in Group I and Group II samples (see Supplementary Tables S5a,b). The difference in mean proportions and the corrected *p*-value of significance are also pointed out.

A Bray–Curtis matrix of samples was used to generate a dendrogram using the UPGMA clustering method. The samples from the five lakes were classified into two groups (Figure 1A). Group I included the four samples from Dianchi Lake and Xingyun Lake. These two lakes were hypertrophic. Group II was a complex cluster, consisting of the six samples from EL, FXL, and LGL. PCoA showed that the classification of lake samples was highly consistent with that by UPGMA analysis (Figure 1B). Furthermore, the PCoA plot indicated that trophic status (eutrophic or mesotrophic-oligotrophic conditions) explained 75.73% of the change in beta-diversity, that was, the total variation in planktonic microbial community structure between groups (PERMANOVA, Pseudo-*F* = 15.867, *p* < 0.01). In addition, compared with the samples in Group II, the samples in Group I had lower taxonomic alpha-diversity (Wilcoxon test, *p* < 0.01) (Figure 1C and Supplementary Table S3).

### Taxonomic Structure of Microbial Communities

Taxonomic annotation showed that the structural composition of the microbial community at the phylum level varied between Groups I and II (Figure 1A and Supplementary Table S4a). We compared the taxonomic structures of these two groups at the phylum-level (Figure 1D and Supplementary Table S5a). Hits to the bacterial phylum Cyanobacteria were more abundant in Group I metagenomic datasets (on average 73.26 ± 13.11%, *q*-value < 0.01, difference in mean proportions [DM] 62.65%) than in Group II datasets, and Actinobacteria were dominant in Group II (on average 28.07 ± 5.15%, *q*-value < 0.05, DM −26.22%). Other notable taxa in Group II were Alphaproteobacteria (on average 15.79 ± 5.32%, *q*-value < 0.05, DM −7.91%), Betaproteobacteria (on average 15.34 ± 2.32%, *q*-value < 0.05, DM −9.50%), Bacteroidetes (on average 10.58 ± 3.07%, *q*-value < 0.05, DM −5.77%), Verrucomicrobia (on average 7.02 ± 4.27%, *q*-value < 0.05, DM −6.77%), Planctomycetes (on average 3.97 ± 3.02%, *q*-value < 0.05, DM −3.00%), and Gammaproteobacteria (on average 3.95 ± 2.78%, *q*-value < 0.05, DM −2.32%).

Moreover, we also identified significant relative abundance differences between the groups at the order-level (Supplementary Figure S4 and Supplementary Table S4b). Of the 209 orders recovered, 16 (7.66%) were overrepresented in one of the two groups (*q*-value < 0.05, absolute difference between means > 1%) (Figure 1D and Supplementary Table S5b). For example, we observed a higher proportion of reads affiliated to Chroococcales (phylum Cyanobacteria, *q*-value < 0.05, DM 73.79%) in Group I than in Group II; conversely, more metagenomic reads of Burkholderiales were detected in Group II than that in Group I (Betaproteobacteria, *q*-value < 0.05, DM −13.37%).

### Functional Structure of Microbial Communities

**FIGURE 2:**
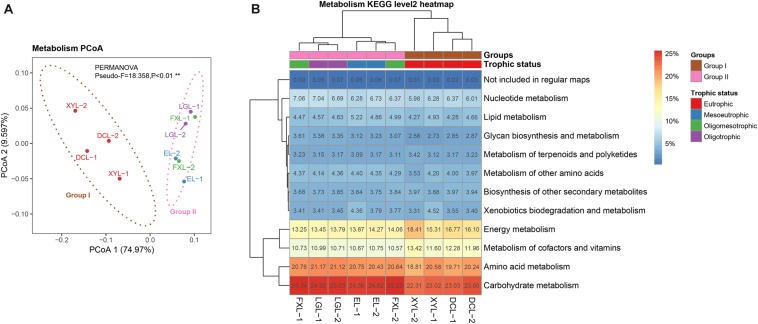
Functional structure of planktonic microbial communities in lakes with different trophic status. **(A)** PCoA based on selected KOs involved in the metabolism pathway with 90% confidence ellipses. **(B)** Heatmap representing the functional clustering of the predicted CDS from the metagenomic data based on the KEGG categories of metabolism level 2. Hierarchical clustering (UPGMA) based on Bray–Curtis dissimilarity matrices.

Assembly of ∼213 Gbps metagenomic sequences yielded ∼14 M contigs (18 Gbps), and ∼24 M predicted CDSs (excluding eukaryotic CDSs) across the five sampled lakes. Between 40.85 and 60.22% of CDSs were assigned to a taxonomic group, and between 20.23 and 27.91% were annotated to KOs (Table 1). PCoA based on selected KOs involved in metabolism revealed a distinct separation of functional structures between Groups I and II (Figure 2A) (PERMANOVA, Pseudo-*F* = 18.358, *p* < 0.01), with a similar ordination pattern to the taxonomic structure (Figure 1B). Interestingly, there was a significant correlation between the functional and taxonomic structures inferred from metagenomic reads (Mantel’s test, Pearson *r* = 0.964; *p* < 0.001).

Carbohydrate metabolism was the most abundant functional category, with relative abundance range from 22.31 to 25.34% within samples (Figure 2B). The second most abundant functional category was amino acid metabolism (18.81–21.17%), followed by energy metabolism (13.25–18.41%). SIMPER analysis was performed to determine the categories making a significant contribution to the differences between groups. Based on the average abundance of functional categories in Groups I and II, we found that functional categories of carbohydrate metabolism, energy metabolism, glycan biosynthesis and metabolism, and metabolism of cofactors and vitamins (SIMPER ratio > 2.0%, FDR padj < 0.01) were different between Group I and Group II samples. A total of 1395 significantly different KOs were successfully mapped onto the KEGG reference metabolic pathway map (Supplementary Figure S5 and Supplementary Table S5c), which indicated that the interrelation of the microbial taxa both within and between these various categories of metabolism deserves further study.

### Correlations Between Environmental Factors and Community Composition

**FIGURE 3:**
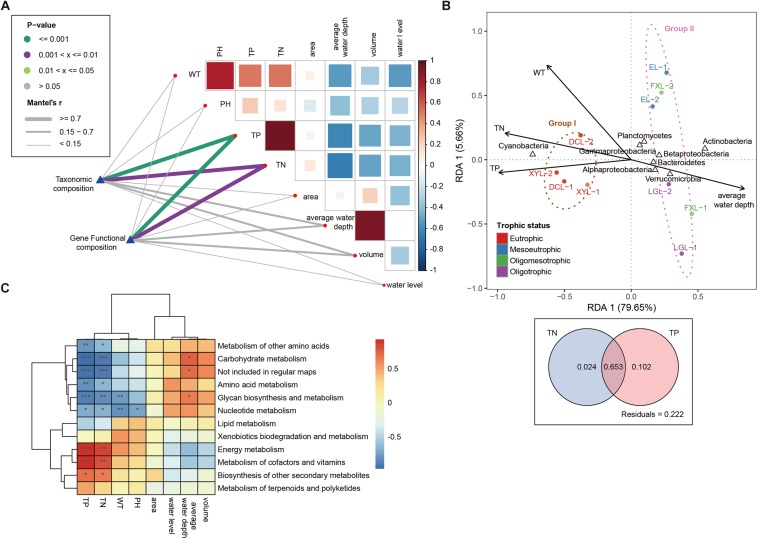
Environmental drivers of community composition. **(A)** Pairwise comparisons of environmental factors are shown. The color gradients and box sizes represent Pearson’s correlation coefficient, and red indicates a positive correlation and blue indicates a negative correlation. Taxonomic and functional (based on metabolism KEGG modules) community composition are related to each environmental factor by Mantel tests. Line width corresponds to the Mantel’s r statistic for the corresponding distance correlations, and line color indicates the statistical significance based on 9999 permutations. **(B)** Redundancy analysis (RDA) is performed on the taxonomic profile (phylum level) and key environmental characteristics (WT, TN, TP, average water depth). Arrows indicate the correlation between environmental parameters and community structure. **(C)** Pearson’s correlations between all environmental factors and the relative abundances of the different metabolism categories (^∗∗∗^*p* < 0.001; ^∗∗^*p* < 0.01; ^∗^*p* < 0.05).

The environmental characteristics of the five lakes are displayed in Table 1. The five lakes involved in this study represented a wide range of trophic status, including oligotrophic, oligo-mesotrophic, meso-eutrophic and eutrophic ecosystems. They range from 0.048 to 5.815 mg/L total nitrogen (TN), and 0.013 to 0.581 mg/L total phosphorus (TP). Furthermore, TN was positively correlated with TP (Pearson’s test *R*^2^ > 0.95, *p* < 0.001) (Figure 3A). Water temperature (WT) and PH were positively correlated with TN and TP, and most of lake geography factors (average water depth, volume and water level) were negatively correlated with lake water quality factors (WT, PH, TN, and TP). It should be noted that the average water depth had a strong negative correlation with WT, TN and TP. Mantel tests indicated that TN and TP were strongly related to taxonomic and gene functional composition (Mantel’s *R* > 0.7, *p* < 0.01) (Figure 3A).

In the RDA model (Figure 3B), environmental factors including TN, TP, WT and average water depth made significant contributions to the relationship between taxonomic composition and environment (*p* < 0.05), and the first axis (RDA1) explained 79.65% of the total variance for the planktonic microbial communities. TN and TP were positively associated with the proportion of Cyanobacteria, but they were negatively associated with the proportion of Actinobacteria, Proteobacteria (Alpha-, Beta-, and Gammaproteobacteria), Bacteroidetes, Planctomycetes and Verrucomicrobia. The results of variation partitioning further showed that TN and TP jointly explained 77.9% of the changes in community structure, among which TN and TP explained 67.7 and 75.5% of the community changes, respectively (Figure 3B). A heatmap showed Pearson’s correlations between all environmental factors and the relative abundances of the functional categories with an important contribution to the differences between groups (Figure 3C). TN and TP were positively related to energy metabolism and metabolism of cofactors and vitamins (*p* < 0.01), while they were negatively related to carbohydrate metabolism and glycan biosynthesis and metabolism (*p* < 0.01).

### Community Metabolic Potential

**FIGURE 4:**
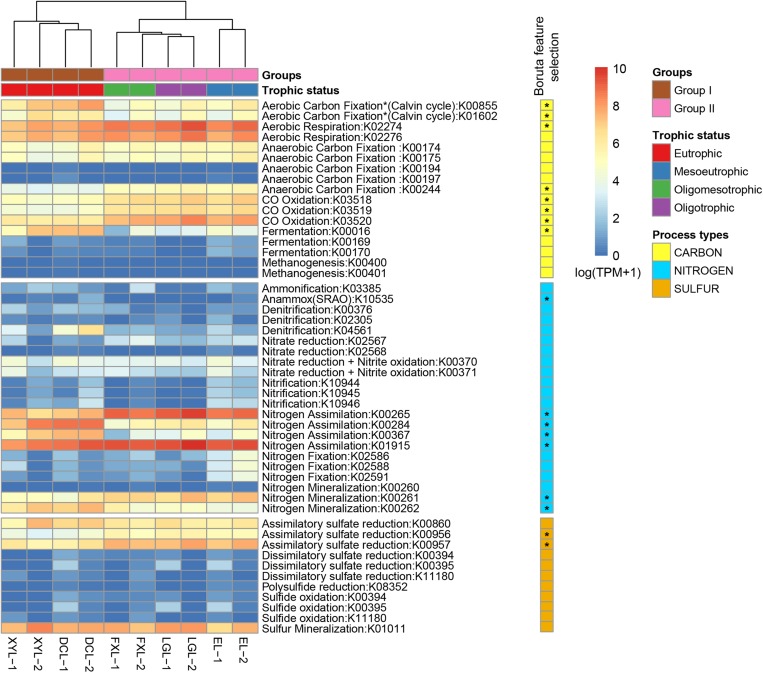
Distribution of KOs involved in C, N, S cycle transformations in samples collected along the five Yun-Gui Plateau lakes. The heatmap displays the relative abundance [log2(TPM + 1)] of KOs across all samples. Hierarchical clustering (UPGMA) based on Bray–Curtis dissimilarity matrices. KOs that differentially segregated across groups are identified by random forest analysis with Boruta feature selection (1000 runs > 4).

We used the TPM value of each marker genes present in the samples from the five lakes as proxies for the genetic potential of microbiota in different steps of the C, N, and S cycles. Among 50 marker genes, 17 were found to have a different distribution between the two groups (Figure 4). Furthermore, marker gene-level hierarchical analysis grouped the samples according to trophic state, which was consistent with the grouping results from other community annotations, including taxonomic classification and metabolism.

**FIGURE 5:**
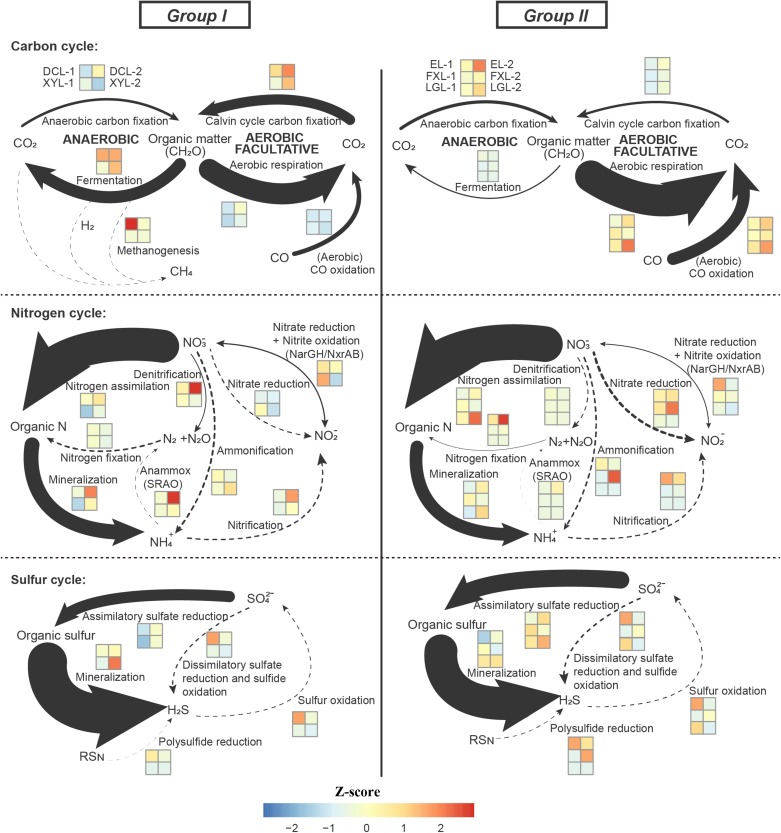
Distribution of genes involved in the carbon, nitrogen and sulfur cycle. Genetic potential for several processes of the C, N, S cycle in the five Yun-Gui Plateau lakes using normalized marked genes. The genetic potentials for each conversion process are assessed based on the combination of these selected marker genes. For marker genes in the same process, the TPM values of genes with the same metabolic function are averaged, and the TPM values of genes with different metabolic functions are added. Arrow sizes are proportional to the genetic potential of the pathways (100% values of each cycle, see Supplementary Table S6). Dotted lines indicate that marked genes are rarely detected. Differences across trophic status are shown by *z*-score heatmap boxes indicated in each C/N/S transformation.

In the carbon cycle, in all lakes, the main pathway detected was aerobic respiration, by Cyanobacteria in Group I, and by Actinobacteria and Alphaproteobacteria in Group II (Group I: 41.84%; Group II: 62.79%; *p* < 0.01) (Figure 5, Supplementary Figure S6, and Supplementary Table S6). Aerobic carbon fixation through the Calvin cycle in Group I was mainly driven by Cyanobacteria, and the potential was higher than that in Group II where it was driven by Cyanobacteria and Betaproteobacteria (*p* < 0.01). In addition, fermentation in Group I was also driven by Cyanobacteria, and the potential was higher than that in Group II where it was driven by Planctomycetes (*p* < 0.01). The potential for CO oxidation in Group II, driven by Actinobacteria and Betaproteobacteria, was higher than that in Group I where it was mediated by Alphaproteobacteria (*p* < 0.01). Notably, low abundance methanogenesis marker genes from Euryarchaeota were detected only in Group I.

In the nitrogen cycle, there was no statistically significant difference in the genetic potential for metabolic processes between the two groups, but we could still observe some interesting results. Marker genes associated with the processes of N assimilation and mineralization accounted for the major proportion of nitrogen cycle genes in both groups (Group I: 65.25% and 29.43%; Group II: 70.87% and 25.34%, respectively) (Figure 5). In communities belonging to Group I, these processes were mainly driven by Cyanobacteria, whereas in Group II they were driven by Actinobacteria. The genetic potential for anammox and denitrification in Group I was higher than that in Group II, while the potential for nitrogen fixation and nitrate reduction in Group I was lower than that in Group II. There was no obvious difference between groups in the potential for, and mediating-microbiota for, ammonification, nitrification, nitrate reduction and nitrite oxidation.

In the sulfur cycle, sulfur mineralization and assimilatory sulfate reduction processes had the highest genetic potential in all lakes. The genetic potential for assimilatory sulfate reduction in Group II was driven by Actinobacteria, and which was higher than that in Group I where it was mediated by Cyanobacteria (*p* < 0.01). Conversely, the potential for sulfur mineralization in Group I, mediated by Cyanobacteria, was higher than that in Group II, where it was mediated by Proteobacteria (*p* < 0.01).

## Discussion

### Distinct Taxonomic Structure and Diversity of Communities in Each Ecosystem

In this study, the percentage of reads that could be taxonomically classified was relatively low. This is a reasonable outcome, explained by the incomplete information contained in reference databases and eukaryotic contamination in environmental metagenomes (Gori et al., 2011; Miller et al., 2019). Nevertheless, the annotation results reflect the composition of the microbial communities in the samples based on high-quality assignments.

Only a few studies have shown that there are remarkable differences in planktonic microbial community structure in freshwater lakes with different trophic status (Dai et al., 2016; Han et al., 2016; Hanson et al., 2017; Ji et al., 2018). In this study, we found that there were large differences in the taxonomic structures of the microbial communities from eutrophic (Group I) and mesotrophic-oligotrophic (Group II, the trophic level from mesotrophy to oligotrophy) freshwater ecosystems in the Yun-Gui Plateau. Moreover, our results suggested that the abundance of the phylum Cyanobacteria (order Chroococcales), which was dominant in eutrophic conditions, was significantly higher in eutrophic environments than that in mesotrophic-oligotrophic environments. In the mesotrophic-oligotrophic ecosystems, the phyla of Actinobacteria and Proteobacteria (Alpha-, Beta-, and Gammaproteobacteria) became dominant, indicating that they have a distinct preference for less eutrophic conditions. Thus, we focused on the correlation between these key taxonomic groups and trophic status. The results of RDA revealed that the occurrence of the phylum Cyanobacteria correlated with trophic status (McMahon and Read, 2013), and the occurrence of Actinobacteria and Proteobacteria (Alpha-, Beta-, and Gammaproteobacteria) with less eutrophic states (Haukka et al., 2006; Ji et al., 2018). The co-occurrence of the key taxa and the particular trophic level indicates that each taxonomic group has unique characteristics in freshwater lake ecosystems. For example, Alphaproteobacteria are competitive in conditions of low nutrient/substrate utilization rate (Newton et al., 2011), and Cyanobacteria outcompete other planktonic microbes for nutrients in eutrophic systems (McMahon and Read, 2013).

Liu et al. (2012) reported that deeper lakes usually have better water quality than shallow lakes, and lake depth plays an important role in explaining the spatial dynamic of water quality in Yunnan Plateau. In our study, we observed the same findings that eutrophic ecosystems were shallow lakes and mesotrophic-oligotrophic ecosystems were deep lakes. It may be due to the deep lakes are associated with higher nutrient dilution ability than shallow lakes (Liu et al., 2012). The correlation analysis between environmental factors indicated that lake depth has significant relationships with TN and TP concentrations. Thus, we propose the average water depth of a lake can be used as a predictor of eutrophication. Additionally, previous studies have reported that the diversity pattern of planktonic bacterial communities in freshwater systems could be significantly correlated with TN and TP concentrations when subjected to eutrophication (Dai et al., 2016; Zeng et al., 2019), and this is consistent with the results of our RDA and variation partitioning. Although there were some differences in sampling time, location and size fraction of samples from the same lake in our study, the clustering of all samples still showed a significant pattern. Samples could be divided into two groups according to the trophic status of the lake. In addition, we observed that there were important differences in taxonomic alpha- and beta-diversity patterns across trophic gradients. Consequently, we conclude that the taxonomic diversity of planktonic microbial communities in freshwater lakes may be related to trophic status. Horner-Devine et al. (2003) observed that the diversity of planktonic bacteria exhibits a downward arched (parabolic) pattern along a gradient of primary productivity. Zeng et al. (2019) also found that the planktonic bacterial community has a positive quadratic relationship with the trophic level. Our results reflected a similar trend, that the alpha-diversity of planktonic microbiota in the eutrophic systems was significantly lower than that in mesotrophic-oligotrophic conditions, and within the mesotrophic-oligotrophic ecosystems, the alpha-diversity in the mesotrophic lake was higher than that in the oligotrophic lake.

### Distinct Functional Structure of Communities in Each Ecosystem

Previous studies suggested that the functional structure of the microbial community is strongly associated with the taxonomic structure across the soil, estuary water and lake ecosystems (Dini-Andreote et al., 2016; Ren et al., 2017; Kieft et al., 2018). The profile of microbial community functions during a Cyanobacterial bloom in a eutrophic freshwater lake has been reported (Steffen et al., 2012; Chen et al., 2018). However, no comparative metagenomics study has been performed revealing the differences in microbial communities in lakes with different trophic status. Using metagenomic analysis, we observed a large difference in the functional structure of the planktonic microbial community between eutrophic and mesotrophic-oligotrophic freshwater ecosystems in the Yun-Gui Plateau lakes, which was strongly correlated with the differences in the taxonomic structures of the communities. By correlation analysis between environmental factors and functional categories, we found that the functional profiles of lakes with different trophic status were mainly correlated to TN and TP concentrations.

Our results showed that genes encoding carbohydrate metabolism and glycan biosynthesis and metabolism were abundant in mesotrophic-oligotrophic freshwater ecosystems, suggesting that microbial communities in surface water of mesotrophic-oligotrophic freshwater ecosystems may have higher utilization rates of organic carbon and higher carbon flux than those of eutrophic systems (Biddanda et al., 2001). Furthermore, genes involved in energy metabolism and cofactors and vitamin metabolism were abundant in the eutrophic ecosystems, which probably related to the high abundance of Cyanobacteria driving rapid energy conversion in this ecosystem and the need for heterotrophic bacteria to produce a large number of cofactors and vitamins (Tang et al., 2010; Li et al., 2018). Accordingly, we inferred that trophic status may contribute to changes in ecosystem function by driving the taxonomic and functional divergence of the microbial community.

### Metabolic Potential of Communities in Each Ecosystem

Owing to variance in the overall functional potential distributions of microbial communities, it can be hypothesized that microbe-mediated biogeochemical cycles are ecosystem-specific, resulting in differences in genetic potential for carbon, nitrogen and sulfur cycling processes in the overlying water of freshwater lakes with different trophic states.

In our study, two high abundance metabolic processes, nitrogen assimilation and nitrogen mineralization, had equal potential across all lakes, indicating that differences in taxonomic composition do not influence the potential of the community to drive these processes. However, the relative abundance of markers of some processes was not constant between ecosystems. For instance, the potential for aerobic respiration and assimilatory sulfate reduction was relatively more abundant in the mesotrophic-oligotrophic freshwater ecosystems, while aerobic carbon fixation, fermentation and sulfur mineralization genes were relatively more abundant in the eutrophic freshwater ecosystems. Although lakes only account for a small fraction of the surface of the Earth (Chen et al., 2015), changes in these processes caused by trophic alteration in freshwater lakes may affect global biogeochemical cycles.

The phylum Cyanobacteria plays a crucial role as a primary producer in freshwater ecosystems, and it provides organic matter through photosynthesis to support the growth of various heterotrophic planktonic bacteria (Fujii et al., 2016). Therefore, it is reasonable that eutrophic ecosystems with a high abundance of Cyanobacteria have a stronger potential for aerobic carbon fixation. Furthermore, in shallow eutrophic lakes, the occurrence of algal blooms in summer not only provides abundant organic matter, but also forms a local dark and anaerobic environment in the overlying water. Stal and Moezelaar (1997) reported that in dark, anoxic conditions, Cyanobacteria use fermentation instead of aerobic respiration as an alternative means of energy generation. Hence, Cyanobacteria in eutrophic ecosystems drive fermentation processes to produce energy to compensate for the relatively low potential of aerobic respiration.

There have been few studies on CO oxidation in lake surface waters. CO in water mainly comes from photochemical degradation of Chromophoric/Colored dissolved organic matter (Stubbins, 2001), which is accelerated by nutrient accumulation (Zhang et al., 2010; Zhou et al., 2018a). Therefore, a eutrophic ecosystem should have more CO flux. However, the abundance of marker genes related to CO oxidation in the mesotrophic-oligotrophic freshwater ecosystems was higher than that in the eutrophic lakes, indicating that the CO oxidation potential in the mesotrophic-oligotrophic lakes was higher. This may be because of microorganisms need more efficient energy harvesting in conditions of low nutrition, and higher primary productivity can reduce the dependence of planktonic microorganisms on exogenous carbon in eutrophic waters. Furthermore, we found that methanogenesis was driven by Euryarchaeota in the eutrophic surface water of Dianchi Lake. Recent works have revealed that a large fraction of CH_4_ oversaturation in aquatic environments is produced in oxygenated surface waters (Townsend-Small et al., 2016; Zhou et al., 2018b). Thus, we suspect that a local anaerobic environment caused by Cyanobacterial blooms in eutrophic lakes may promote the production of CH_4_ in aerobic overlying water to some extent (Xing et al., 2012). Evans et al. (2017) reported that eutrophication causes lakes to transition from sinks to sources of carbon. Our data suggest carbon accumulation in the eutrophic lake because of increased carbon fixation potential relative to respiratory potential.

Wu et al. (2019) found that algal blooms could accelerate the nitrogen cycling rate. Our results showed that there was no dramatic divergence in the potential for N-cycle processes between the eutrophic and the mesotrophic-oligotrophic freshwater ecosystems, but there were some noteworthy differences in anammox, denitrification and nitrogen fixation. Rich organic matter produced by algal blooms can be converted into ammonia and nitrate for anammox and denitrification (Wu et al., 2019). Hence, we infer that there are high potentials for these two processes in eutrophic ecosystems, which may be the result of an accelerated N-cycle within this ecosystem. In addition, the lower potential for nitrogen fixation in eutrophic ecosystems is the result of the presence of rich organic matter, while the higher potential for nitrogen fixation in the mesotrophic-oligotrophic ecosystems is most likely related to a lack of organic matter.

Although sulfur cycling in freshwater sediments and vertical water columns has been well studied (Cai et al., 2019; Ren et al., 2019), the genetic potential for sulfur transformation in surface waters of lakes with different trophic status has not been studied. With the death of a large number of Cyanobacteria in eutrophic lakes, the high content of sulfur-containing amino acids in their cells might be released (Lu et al., 2013), resulting in a water column enriched with organic sulfur. Our results showed that planktonic microbial communities in the eutrophic ecosystems exhibited less abundance of assimilatory sulfur reduction-related genes to produce organic sulfur; on the contrary, planktonic microbial communities in the eutrophic ecosystems exhibited higher potential for sulfur mineralization than those in the mesotrophic-oligotrophic environments, which may lead to the tendency of the eutrophic ecosystems to release H_2_S gas.

## Conclusion

Our research reports on the planktonic microbial communities of five plateau freshwater lakes with different trophic status, located in Yunnan, China. The trophic alterations caused by anthropogenic activities are not only related to microbial community composition, but also to the genetic potential for important carbon, nitrogen and sulfur biogeochemical cycling reactions mediated by microbes in the surface waters.

The overall differences in metabolic functions and the genetic potential for elemental cycling were strongly related to divergence in the taxonomic structure and diversity of the planktonic microbial communities. Energy metabolism and cofactors and vitamin metabolism had strong representation in the eutrophic ecosystems; carbohydrate metabolism and glycan biosynthesis and metabolism had strong representation in the mesotrophic-oligotrophic ecosystems. Moreover, the phylum Cyanobacteria, dominant in the eutrophic ecosystems, mainly mediated the processes of aerobic respiration, fermentation, nitrogen assimilation, nitrogen mineralization, assimilatory sulfate reduction and sulfur mineralization in this system. The phyla Actinobacteria and Proteobacteria (Alpha-, Beta-, and Gammaproteobacteria), Verrucomicrobia and Planctomycetes showed higher relative abundance in the mesotrophic-oligotrophic ecosystems than those in the eutrophic ecosystems. In the mesotrophic-oligotrophic ecosystems, aerobic respiration, nitrogen assimilation, nitrogen mineralization and assimilatory sulfate reduction were mainly mediated by the phylum Actinobacteria, sulfur mineralization was mainly driven by Alphaproteobacteria, and fermentation was mainly driven by Planctomycetes. Planktonic microbial communities in the eutrophic ecosystems had higher potential for aerobic carbon fixation, fermentation, methanogenesis, anammox, denitrification and sulfur mineralization than those in the mesotrophic-oligotrophic ecosystems. Besides, planktonic microbial communities in the mesotrophic-oligotrophic ecosystems had higher metabolic potentials for aerobic respiration, CO oxidation, nitrogen fixation and assimilatory sulfate reduction than those in the eutrophic ecosystems. Overall, trophic preference of some key taxonomic groups leads to communities with distinct taxonomy and functions, corresponding to ecosystem-specific carbon, nitrogen and sulfur cycles in Yun-Gui Plateau freshwater lakes characterized by different trophic status.

## Data Availability Statement

Metagenomics data sets have been deposited in the NCBI Short Read Archive (SRA) the Bioproject Number PRJNA548910 (accessions: SAMN12058482, SAMN12058483, SAMN12058484, SAMN12058485, SAMN12058486, SAMN120-58487, SAMN12058488, SAMN12058489, SAMN120-58490, SAMN12058491).

## Author Contributions

MS carried out the library preparation, sample sequencing, data analysis, and wrote the first draft of the manuscript. QL assisted in library preparation and sample sequencing. MR polished the manuscript. YL and JW helped the revision of the manuscript. LC performed the sample collection and filtration, and polished the manuscript. TL and JZ conceived the study and designed the experiments. All authors read and approved the final manuscript.

## Conflict of Interest

The authors declare that the research was conducted in the absence of any commercial or financial relationships that could be construed as a potential conflict of interest. The reviewer JW declared a shared affiliation, with no collaboration, with one of the authors, MR, to the handling Editor at time of review.

## Footnotes

1 https://github.com/shenmengyuan/Yun-Gui_plateau_lake

2 www.kegg.jp/ghostkoala/

3 https://pathways.embl.de/
