# Supplementary material for: Trophic Status Is Associated With Community Structure and Metabolic Potential of Planktonic Microbiota in Plateau Lakes
Source: Front Microbiol. 2019 Nov 7;10:2560. doi: 10.3389/fmicb.2019.02560 (PMC6853845; doi:10.3389/fmicb.2019.02560)
Supplement: Supplementary file 1 [file Data_Sheet_1.PDF]

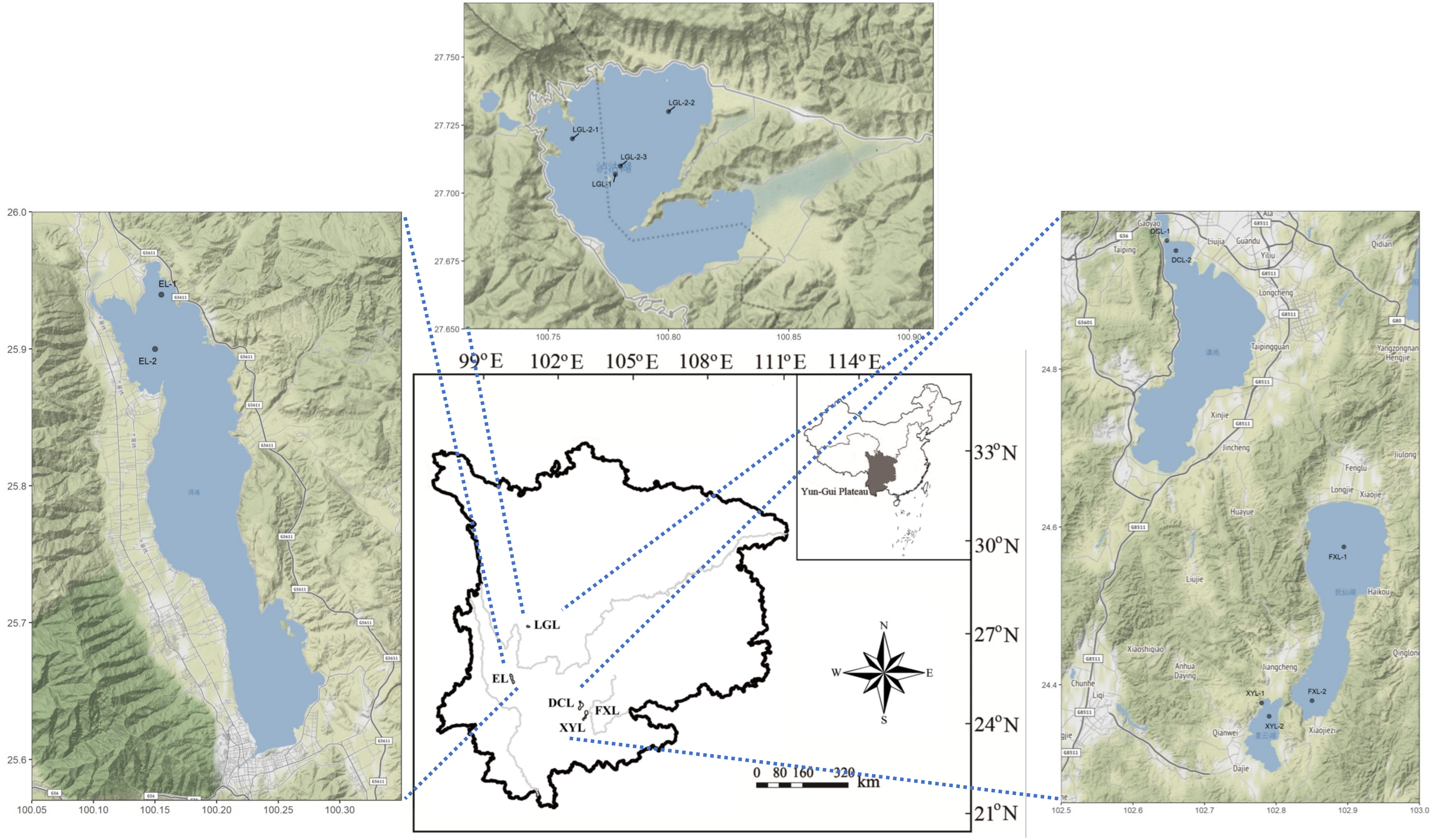


**Figure S1.** The geographical locations of the five Yun-Gui Plateau Lakes. Map showing the location of the five studied lakes. DCL, Dianchi Lake; FXL, Fuxian Lake; EL, Erhai Lake; LGL, Lugu Lake; XYL, Xingyun Lake.

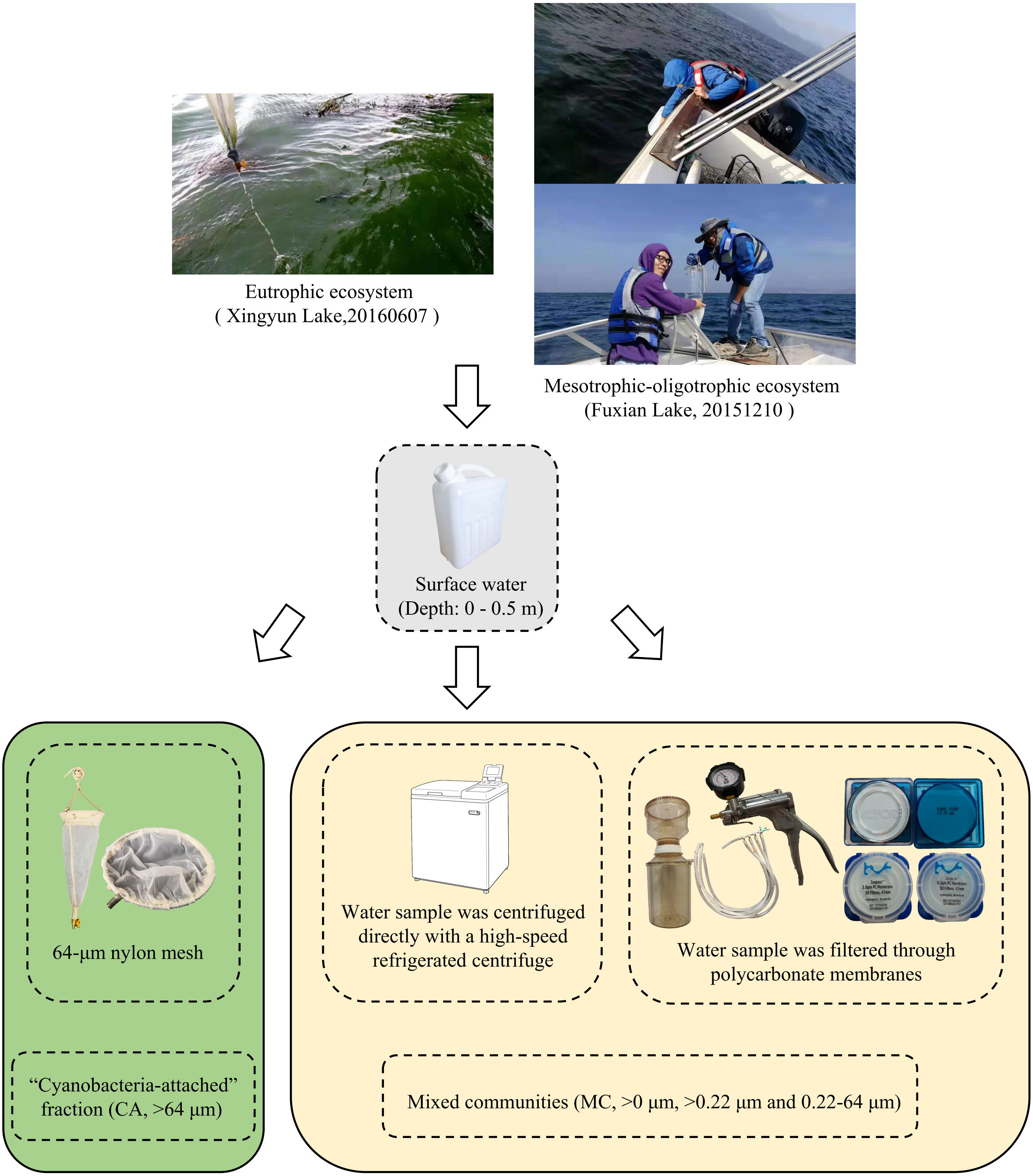


**Figure S2.** Schematic diagram of different methods for biomass collection of microbial communities from eutrophic and mesotrophic-oligotrophic ecosystems. Some field-of-view photos during sampling were shown. The "Cyanobacteria-attached" fraction (CA, >64 μm) was concentrated by using a 64-μm nylon net in green rectangle. The collection of mixed communities used centrifugation and filtration methods  in yellow rectangle. The centrifugation method enriches all microorganisms, while the filtration method collects more than 0.2 μm.

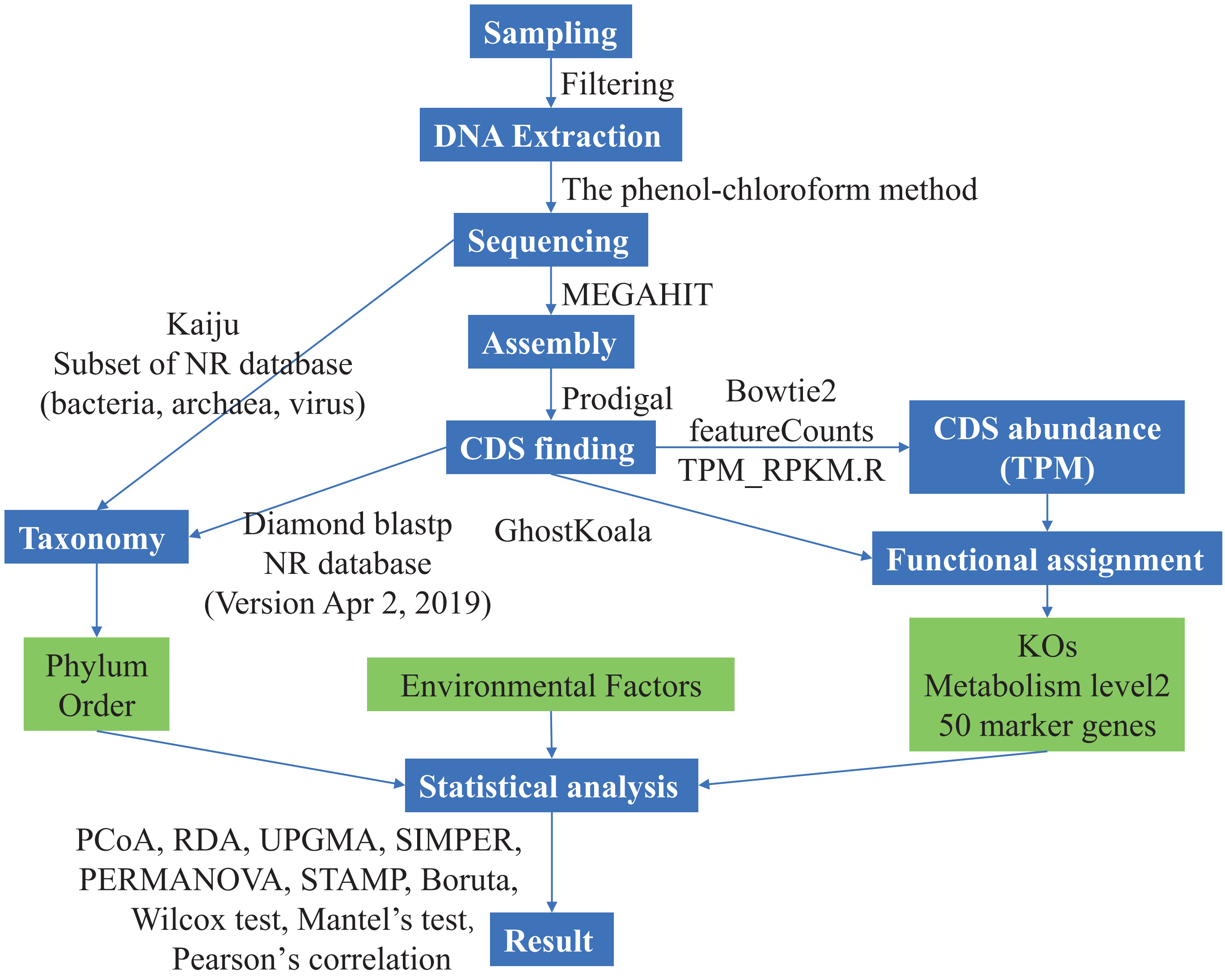


**Figure S3.** An overview of analysis pipeline to metagenome of each lake samples at different trophic levels, including the steps of sampling, DNA Extraction, sequencing, assembly, CDS finding, CDS abundance calculating, taxonomy, functional assignment and statistical analysis.

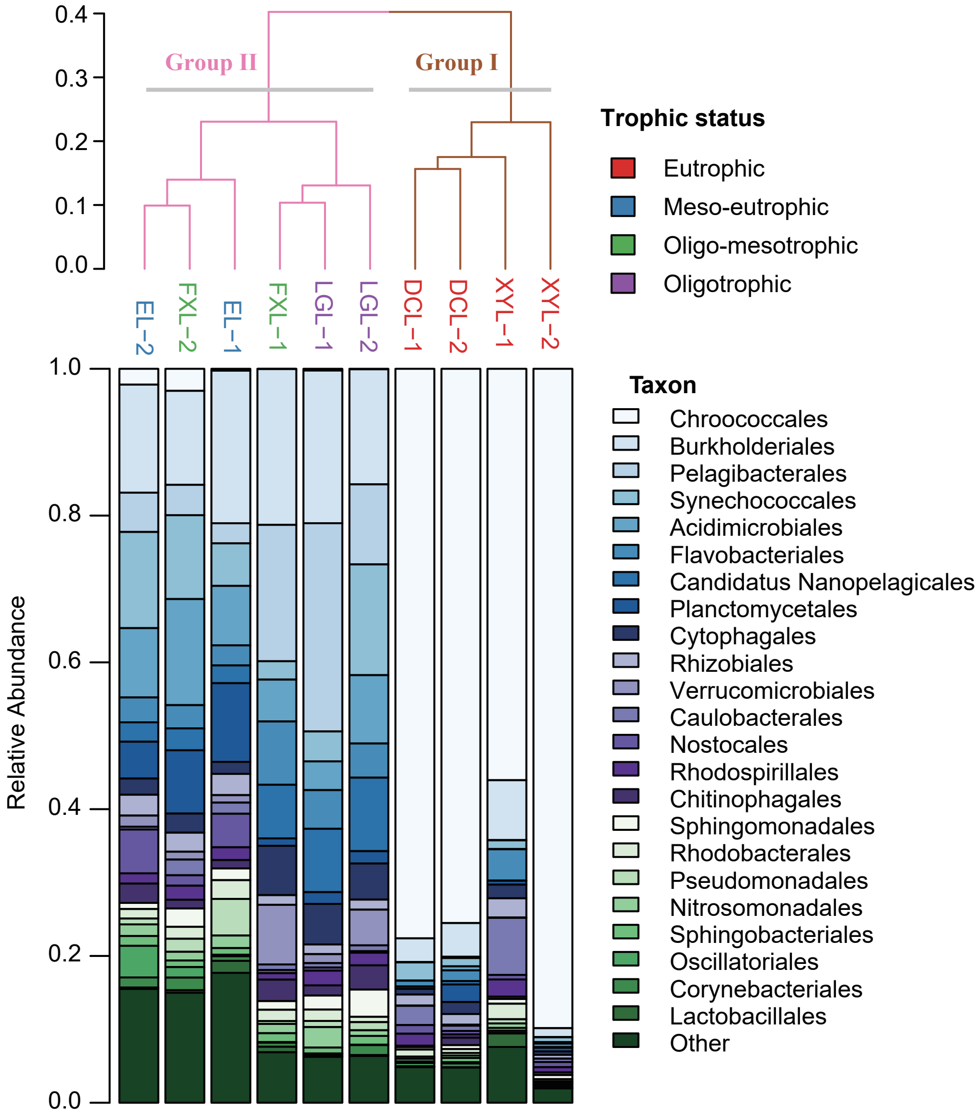


**Figure S4.** Taxonomic community structure across samples. The relative abundance of reads grouped at order-level lineages. Orders with <0.5% relative abundance are shown as “Other”. Hierarchical clustering (UPGMA) based on Bray-Curtis dissimilarity matrices. See detailed information in Supplementary Table S4b.

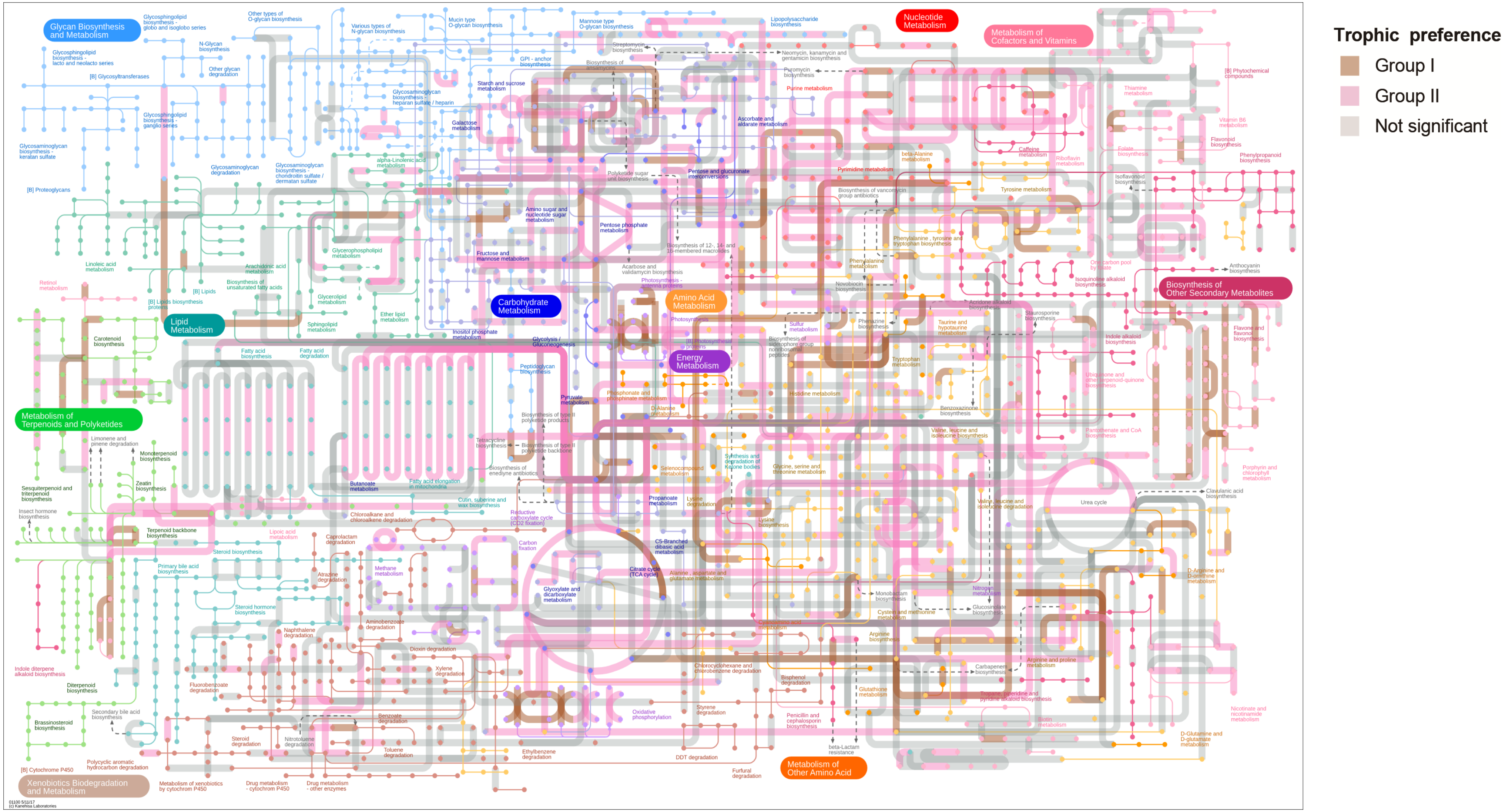


**Figure S5.** Metabolic pathways of trophic preference KO lists. The color of Group I preference pathway is brown, and the color of Group II preference pathway is plink, and the color of insignificant preference pathway is gray.

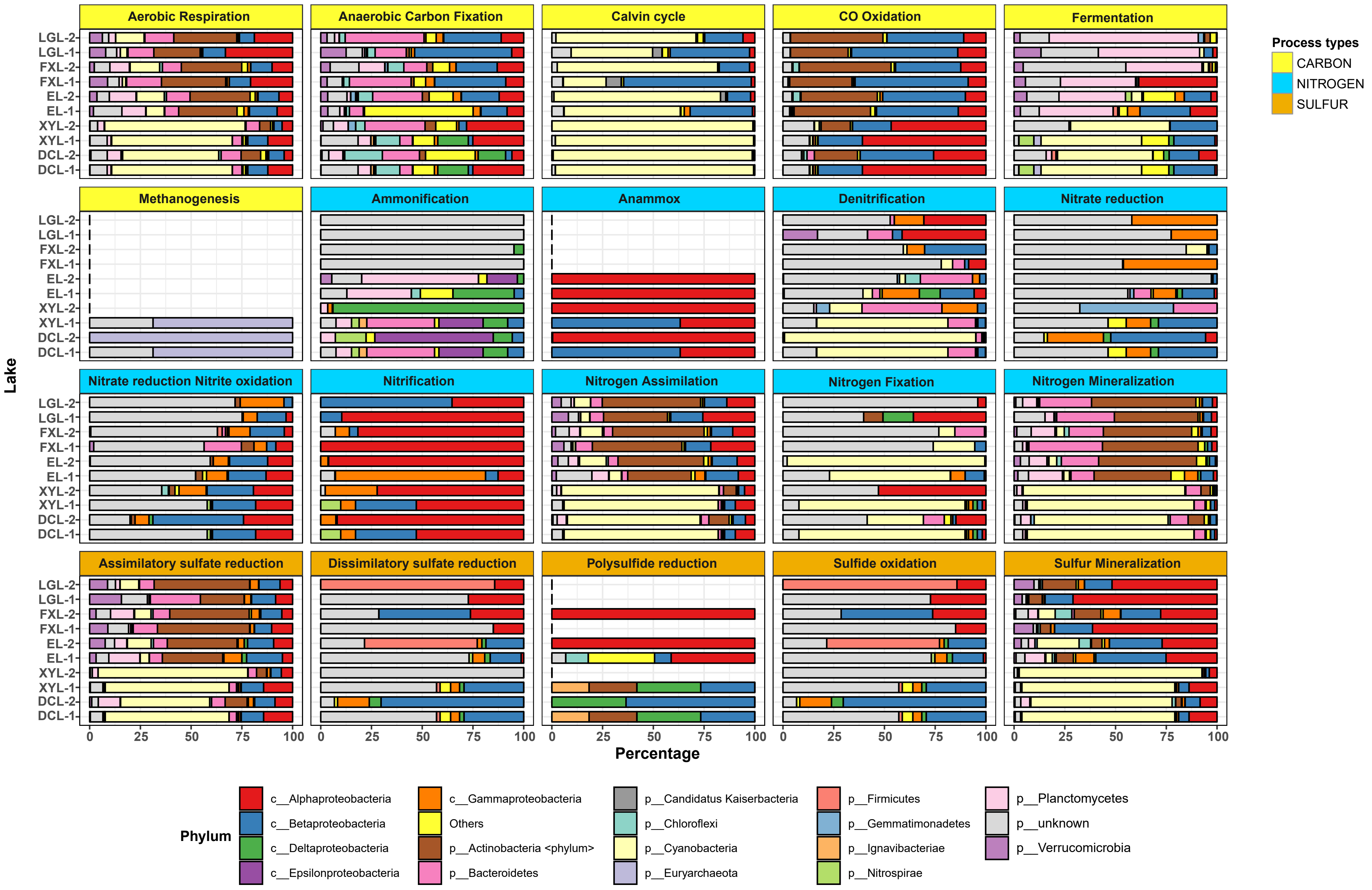


**Figure S6.** Relative distribution of taxonomic groups putatively involved in major carbon, nitrogen and sulfur cycles in five Yun-Gui plateau lakes inferred from metagenomic data. The x-axis of each figure indicates the relative abundance of specific pathways associated with given taxonomic groups, and the y-axis indicates the samples with different trophic gradient.
