# Supplementary material for: Trophic Status Is Associated With Community Structure and Metabolic Potential of Planktonic Microbiota in Plateau Lakes
Source: Front Microbiol. 2019 Nov 7;10:2560. doi: 10.3389/fmicb.2019.02560 (PMC6853845; doi:10.3389/fmicb.2019.02560)
Supplement: Supplementary file 2 [file Table_1.DOCX]

| **Table S1.** The detailed collection method of ten microbial samples used in this study. | | | |
| --- | --- | --- | --- |
| **Sample ID** | **Size fraction of community (μm)** | **Water volume** | **Description** |
| DCL-1 | >64 | 20L | The microbial samples collected from lakes were enriched by 64-μm nylon mesh; |
| DCL-2 | >0.2 | 250mL | Lake water was pre-filtered through 10.0-µm and 3.0-µm filters, and then filtered onto 0.22-µm pore size polycarbonate membranes; |
| XYL-1 | >64 | 20L | Same as DCL-1; |
| XYL-2 | >0.2 | 400mL | Same as DCL-2; |
| EL-1 | >0 | 7L | Lake water was centrifuged directly with a high-speed refrigerated centrifuge, and the products at the bottom of the centrifuge tube were scraped for preservation (about 60mg); |
| EL-2 | >0.2 | 1L | Same as DCL-2; |
| FXL-1 | 0.2-64 | 10L | Lake water was pre-filtered through 64-μm nylon mesh, then cells were further filtered with and collected on 0.22-μm filters; |
| FXL-2 | >0.2 | 2.5L | Same as DCL-2; |
| LGL-1 | >0.2 | 20L | Lake water was directly filtered through a filter with a pore size of 0.22-µm. |
| LGL-2 | >0.2 | 5L | Same as DCL-2; |
| The 10.0-µm,3.0-µm and 0.2-µm pore size polycarbonate membranes were both 47 mm diameter, EMD Millipore, Billerica, MA, United States | | | |
|  |  |  |  |
